# Physical Mapping of QTLs for Root Traits in a Population of Recombinant Inbred Lines of Hexaploid Wheat

**DOI:** 10.3390/ijms241310492

**Published:** 2023-06-22

**Authors:** Xiaoqing Li, Anton P. Wasson, Alexander B. Zwart, Alex Whan, Peter R. Ryan, Kerrie Forrest, Matthew Hayden, Sabrina Chin, Richard Richards, Emmanuel Delhaize

**Affiliations:** 1 CSIRO Agriculture and Food, Canberra, ACT 2601, Australia; anton.wasson@csiro.au (A.P.W.); alec.zwart@data61.csiro.au (A.B.Z.); alex.whan@csiro.au (A.W.); peter.ryan@csiro.au (P.R.R.); richard.richards@csiro.au (R.R.); 2 Agriculture Victoria Research, AgriBio, Centre for AgriBioscience, Bundoora, VIC 3083, Australia; kerrie.forrest@agriculture.vic.gov.au (K.F.); matthew.hayden@agriculture.vic.gov.au (M.H.); 3 School of Applied Systems Biology, La Trobe University, Bundoora, VIC 3086, Australia; 4 Department of Botany, University of Wisconsin, Madison, WI 53706, USA; schin7@wisc.edu; 5 Australian Plant Phenomics Facility, Research School of Biology, The Australian National University, Canberra, ACT 2601, Australia; manny.delhaize@anu.edu.au

**Keywords:** bread wheat, root morphology, traits, genetics, candidate genes

## Abstract

Root architecture is key in determining how effective plants are at intercepting and absorbing nutrients and water. Previously, the wheat (*Triticum aestivum*) cultivars Spica and Maringa were shown to have contrasting root morphologies. These cultivars were crossed to generate an F_6:1_ population of recombinant inbred lines (RILs) which was genotyped using a 90 K single nucleotide polymorphisms (SNP) chip. A total of 227 recombinant inbred lines (RILs) were grown in soil for 21 days in replicated trials under controlled conditions. At harvest, the plants were scored for seven root traits and two shoot traits. An average of 7.5 quantitative trait loci (QTL) were associated with each trait and, for each of these, physical locations of the flanking markers were identified using the Chinese Spring reference genome. We also compiled a list of genes from wheat and other monocotyledons that have previously been associated with root growth and morphology to determine their physical locations on the Chinese Spring reference genome. This allowed us to determine whether the QTL discovered in our study encompassed genes previously associated with root morphology in wheat or other monocotyledons. Furthermore, it allowed us to establish if the QTL were co-located with the QTL identified from previously published studies. The parental lines together with the genetic markers generated here will enable specific root traits to be introgressed into elite wheat lines. Moreover, the comprehensive list of genes associated with root development, and their physical locations, will be a useful resource for researchers investigating the genetics of root morphology in cereals.

## 1. Introduction

Root morphology plays a major role in how efficiently plants take up water and mineral nutrients [1,2,3]. The root system of monocotyledons is comprised of different root types [4]. For instance, hexaploid wheat (*Triticum aestivum*) has four to six primary or seminal roots that develop from the embryo and many nodal roots that develop at later stages from the crown of the main stem and tillers just below the soil surface [4]. The seminal roots of wheat are typically thinner than the nodal roots and grow deeper, whereas the nodal roots have a wider growth angle and tend to explore more of the shallower soil layers. Both seminal and nodal roots form primary and secondary lateral roots which ultimately account for most of the total root length in mature plants.

While many aspects of root structure are under genetic control, root growth also responds to environmental signals. For instance, soil compaction or the onset of water stress or nutrient deficiency can trigger specific changes to the relative growth of the different root types, which alter overall root morphology in order to ameliorate the stress [5,6]. There is no single root morphology that is ideal for crops grown under all conditions because it depends on the availability of water and nutrients and their distribution down the soil profile. The root structure best suited for one environment may be poorly suited for another environment. For example, root systems that efficiently absorb deep water and highly mobile nutrients such as nitrate or potassium will differ from those better suited for accessing surface moisture and sparingly mobile nutrients like phosphate which tends to concentrate near the soil surface [7]. Therefore, the “steep, cheap and deep” root ideotype that is so useful for intercepting deep water and mobile nutrients [8] would be a disadvantage when phosphate is limited or rainfall events are short and regular.

Wheat is a major global food crop that contributes some 20% of the calories consumed by humans [9]. It is grown worldwide and has adapted to highly contrasting climatic conditions and agricultural production systems. With the continuing drive to improve yields and to adapt to climate change, defining the most effective root system for a specific region is becoming increasingly important. Identifying molecular markers and genes associated with specific root traits will enable breeders to develop germplasm better suited to particular conditions. We undertook a genetic analysis of recombinant inbred lines (RILs) derived from a cross between Spica and Maringa—two hexaploid wheat genotypes with highly contrasting root traits [10]. The RILs were grown in soil and scored for seven root traits and two shoot traits. Flanking markers from the resulting quantitative trait loci (QTL) were located on the physical map of the Chinese Spring reference genome and compared with the QTL identified in previous studies on root traits of wheat. To determine if previously cloned genes from wheat and other monocotyledonous species were encompassed by the QTL, we located the genes on the Chinese Spring physical map. The Chinese Spring reference genome provides researchers with a powerful tool to identify genes that underlie QTLs in order to accelerate breeding efforts [11].

## 2. Results

**Figure 1:**
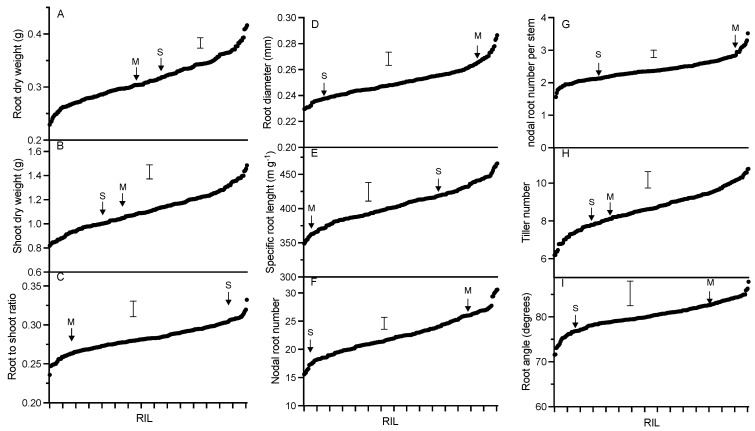
Distribution profiles of various traits across RILs. BLUP data with SE as error bars and locations of parental lines are shown (M = Maringa and S = Spica).

A population of RILs generated from Spica and Maringa was scored for seven root-related phenotypes and two shoot-related phenotypes. The heritability ranged between 0.62 and 0.80 for all traits, except for the root angle which had a heritability of 0.33 (Table 1). The distribution of values of the RILs exceeded the difference between parental lines for all nine traits with root dry weight, shoot dry weight and tiller number showing the greatest transgressive variation (Figure 1). When the variation of all the QTLs for each trait was summed together, they explained between 23 to 72% of the total variation (Table 2).

**Figure 2:**
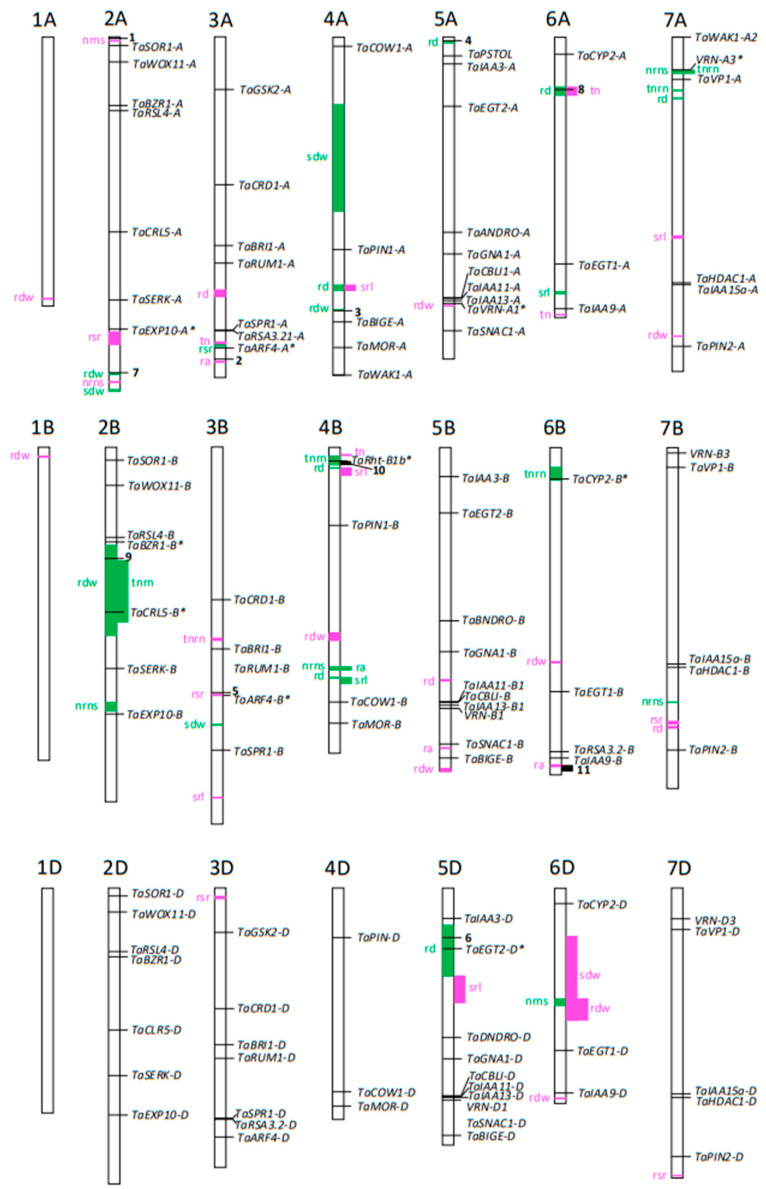
Schematic of positions of QTLs on the Chinese Spring IWGSC v1.0 physical map along with the previously identified QTLs and cloned genes associated with root development in wheat and other species. Chromosomes and locations are drawn approximately to scale. Boxes and letters in green denote a QTL with LOD scores greater than 3.0 while boxes and letters in magenta denote a QTL with LOD scores less than 3.0. Previously cloned root genes associated with root morphology in wheat and other monocotyledons are shown on the right side of the chromosomes with horizontal lines indicating locations on the chromosomes. Since the RILs were segregated for the semi-dwarfing gene *Rht1-B1b*, this gene is included in the Figure. Gene names with asterisks (*) denote genes that are inside a QTL or within 5 Mb of a flanking marker of a QTL identified in our study. Locations shown by **bold numbers** indicate locations of candidate genes or SNPs identified from other studies that are inside QTL or within 5 Mb of the closest flanking marker identified in our study. Numbers 1–6 correspond to QTL with candidate wheat genes as identified by the meta-analysis of Soriano and Alvaro [12]: 1; *Root_MQTL_17 TraesCS2A01G004600*, 2; *Root_MQTL_35 TraesCS3A01G477800*, 3; *Root_MQTL_45 TraesCS4A01G306600*, 4; *Root_MQTL_55 TraesCS5A01G012600,* 5; *Root_MQTL_40 TraesCS3B01G334000*, 6; *Root_MQTL_63 TraesCS5D01G096700*. Numbers 7–9 correspond to SNPs significantly associated with root QTL as identified in a GWAS by Ma et al. [13] including SNPs on 2B and on 6A for average root diameter and a SNP on 2A for total root area/volume. Root QTLs identified by Yang et al. [14] from a biparental QTL analysis that overlap with our QTL are shown as black boxes and are numbered at locations on chromosome 4B (10; root dry weight and root to shoot ratio) and chromosome 6B (11; root length and shoot dry weight). The traits we analyzed included the root dry weight (rdw), shoot dry weight (sdw), root-to-shoot ratio (rsr), root diameter (rd), specific root length (srl), tiller number (tn), total nodal root number (tnrn), nodal root number per stem (nrns) and root angle (ra).

Between four to twelve QTLs were identified for each trait (Table 2). These were divided into those with LOD scores greater than 3.0 (Table 3) and those with LOD scores less than 3.0 (Table 4). For QTL with LOD scores greater than 3.0, Table S1 shows the genetic locations of the flanking markers in cM, their LOD scores, additive effect, and the percentage of variation that they explain. Table 3 shows the physical locations of these markers on the Chinese Spring reference genome where the traits were mapped to genomic intervals ranging from 0.22 Mb to 230 Mb. Similarly, Table S2 lists the genetic locations of QTLs with LOD scores less than 3.0, and Table 4 shows their physical locations with intervals ranging from 0.03 to 190 Mb. A schematic diagram of the chromosomes summarises the physical locations of the QTL and shows that some traits had overlapping QTL (Figure 2). The co-location of some QTLs was expected (such as the total nodal root number and nodal root number per stem), whereas the co-location of other traits was not expected and the connection was less obvious (e.g., nodal root number per stem and root-to-shoot ratio).

We searched for candidate genes that underlie the QTL by determining if any previously cloned genes from wheat or other monocotyledons associated with root structure co-located with the QTL on the physical map. A list of previously cloned genes was assembled and located on the Chinese Spring reference genome. These genes were categorised into those that had been identified from wheat and those identified from other monocotyledons (Table S3). For any given candidate gene, the locations of homeologs from the three wheat genomes were also included. Table S3 provides the gene names, plant species, and their physical locations on the wheat genome. For genes associated with root development in species other than wheat, the closest three homologs in the wheat genome were found and these were located on the physical map. Figure 2 provides a visual representation of the overlaps between QTLs and genes on the chromosomes. Also shown in Figure 2 are the physical locations of candidate genes and QTLs identified in previous studies that either overlap or are within 5 Mb of the QTL from our study.

## 3. Discussion

This study identified over 60 QTLs for various root traits with each trait controlled by up to seven QTLs with LOD greater than 3.0 (Table 2). When summed together the QTLs explained more than 40% of the total variation for each trait with the exception of the tiller number which was ~23%. On three occasions, the QTL for the root diameter co-localised or overlapped with the QTL for specific root length (*Qrdw-4A* and *Qsrl-4A*; *Qrd-4B1* and *Qsrl-4B2*; *Qrd-4B2* and *Qsrl-4B1*). The association of the root diameter with the specific root length is logical since root thickness would be a key driver of the specific root length. Similarly, QTLs for nodal root number per stem and total nodal root number were co-located, but only in one instance, (*Qnrns-7A* and *Qtnrn-7A2*) indicating that these parameters are not always related. Interestingly, the QTL of the tiller number did not co-locate with the that of the total nodal root number, indicating that these traits are controlled by independent genes.

Determining the physical locations of QTLs is valuable for identifying candidate genes but care is required since the genomes of the parental genotypes in QTL studies can differ markedly from the Chinese Spring reference genome. Genomic rearrangements as well as the abundance and location of transposable elements, often differ between genotypes. Nevertheless, physical mapping is an important early step toward cloning target genes by helping to identify the genomic regions where causative genes are located. Mapping the physical locations of QTLs on the Chinese Spring reference genome allowed us to establish the sizes of QTLs as defined by flanking markers. Importantly, it allowed previously cloned genes, with roles in root development, to be identified as candidates controlling these traits. Establishing the physical locations of QTLs also enabled different studies to be directly compared with one another more rigorously than simply relying on genetic distances. QTL studies rarely use the same genotypes and past QTL studies often used markers that have not been mapped to physical locations on the Chinese Spring reference genome. To overcome these limitations, Soriano and Alvaro [12] undertook a meta-analysis of root-related QTLs from thirty-one biparental wheat populations using a consensus map for various marker types. That study provided gene models based on IWGSC v1.0 to identify candidates that underlie the QTL for several root traits which allowed us to establish which QTL in past studies likely overlapped with the QTL identified in our study. We identified six candidate genes from the meta-analysis of the Soriano and Alvaro [12] study that were either inside or within 5 Mb of the QTL identified in our study (Figure 2). The QTLs derived from the meta-analysis were often not for the same root trait, and this was also apparent for QTLs from more recent studies that overlapped with those of our study (Figure 2). For example, often the QTLs were for apparently unrelated traits such as root angle and root dry weight, whereas other QTLs were related or the same trait. Examples of similar traits are the QTL for root diameter mapped by Ma et al. [13] that co-located with *Qrd-6A* for root diameter in our study, and the QTL for total root area/root volume mapped by Yang et al. [14] that is located close to a QTL for root dry weight (*Qrdw-2A*; Figure 2).

A list of the physical locations of wheat genes previously associated with root development enabled us to determine whether any of these genes were located within the QTL from our study. The list not only includes genes cloned from wheat but also the homologs in wheat of genes cloned from other monocotyledons (Table S3). The evidence that all these have a role in root development varies from compelling (supporting evidence from both mutants and transgenic lines; [15]) to suggestive (phenotypes generated in transgenic lines by over-expression of genes; [16]). The list provides a quick reference guide for researchers to locate candidate genes for QTL and mapping studies. It was a useful tool for the present study and should also be a valuable resource to future researchers for investigating the genetics of root morphology in wheat.

We found several candidate genes that either co-located or were within 5 Mb of our QTL (Figure 2). For example, the rice *CLR5* gene controls crown root development [17] and the wheat homolog on chromosome 2B, *TaCLR5-B*, is located within a QTL for total nodal root number and root dry weight and can be considered a strong candidate controlling those traits. Similarly, *VRN-A1* is a candidate gene underlying the *Qrdw-5A* for root dry weight on chromosome 5A because, even though it lies just outside a flanking marker, it has previously been shown to affect various aspects of root morphology in wheat [18]. Interestingly, *VRN-A3* on chromosome 7A was located near QTLs for total nodal root number and nodal root number per stem (Figure 2) but there is currently no other evidence to show this gene plays a role in root morphology of wheat. Other candidate genes also do not have obvious roles in controlling the traits. For instance, it is not clear how *TaEXP10-A*, which encodes an expansin protein on chromosome 2A, controls the root-to-shoot ratio (*Qrsr-2A*; Table 4) when the homolog in rice controls root cell expansion [19]. Similarly, *TaEGT2-D* was located near the middle of a QTL for root diameter on chromosome 5D (*Qrd-5D*; Table 3), whereas that gene in barley and wheat specifically controls the root angle [20]. *TaCLR5-B* and *VRN-A1* as well as *TaARF4-A* and *TaCYP2-B* (two additional genes co-locating with QTL on chromosomes 3A and 6B, respectively), encode transcription factors or other regulatory proteins, and it is conceivable that alleles of these genes have multiple effects on root morphology. Interestingly, the semi-dwarfing gene *TaRht-B1b* was located within a QTL for the total nodal root number on chromosome 4B (Figure 2). The RILs segregated for *TaRht-B1b* but the gene has not been previously identified as specifically controlling nodal root number. Indeed, when grown with adequate P fertiliser, a semi-dwarf line with *TaRht-B1b* did not differ for a range of root attributes when compared to an isoline with the wild-type *Rht-B1a* allele [21]. A study assessing the effects of *Rht* genes on roots also found that the semi-dwarfing alleles did not affect root morphology including length and biomass for bread wheat grown in a variety of experimental conditions including the field [22]. Similarly, a study conducted on bread wheat genotypes commonly used in the Great Plains region of the USA found that the semi-dwarfing alleles did not affect the growth of root systems [23]. By contrast, the *Rht-B1b* allele significantly reduced root biomass in durum wheat [24], illustrating the importance that genetic background can have on gene effect. It should be noted that genes can co-locate with a QTL by chance, which emphasises the importance of providing additional experimental evidence to verify whether the candidate genes influence the trait in question.

Several traits were mapped to small regions on the physical map that encompassed only a few genes. The most precisely mapped QTLs were those for root dry weight which mapped to intervals of only 0.22 Mb (*Qrdw-4A*: Table 3) and 0.03 Mb (*Qrdw-5A*: Table 4). *Qrdw-4A* encompasses eight genes that are predicted with high confidence on the reference genome but none of these encode proteins with obvious roles affecting root morphology. *Qrdw-5A* encompasses the two genes *TraesCS5A02G396900* and *TraesCS5A02G397000* which encode an uncharacterised protein and a putative protein involved in a replication complex. As noted above, *VRN1-A* was found near a flanking marker (within 4 Mb) of *Qrdw-5A* so is considered a more likely candidate gene for this QTL.

Climate change is already affecting rainfall patterns and the root systems that are best adapted to particular regions may change. In areas where stored water is important, root systems that are able to access deep moisture prior to grain filling will be most beneficial. For example, alleles of the *Dro1* gene of rice control the rooting depth by altering the angle of the crown roots [25]. Plants with a narrow rooting angle grew deeper and yielded better in drought conditions than genotypes with shallower roots. By contrast, plants with alleles of *Dro1* that confer more shallow root systems were more efficient at accessing P since most of that resource was localised near the soil surface [26]. We did not find homologs of *Dro1* that co-located with the QTL for the root angle in our study. However, the important influence of root angle has been confirmed in durum wheat (*T. turgidum*) where genotypes with steep and deep roots yielded better under drought conditions than shallow-rooted genotypes. Conversely, the shallow-rooted genotypes performed better under well-watered conditions [27]. These observations emphasise the point that one root morphology does not fit all situations. Instead, different root morphologies can be better suited to specific environments.

We mapped the QTLs for root traits in wheat using parents with contrasting root morphologies. RILs are useful experimental germplasm for assessing the value of a given trait because the tails of the population for each trait can be compared in field trials to assess their value under various environmental conditions. Mapping the flanking markers onto the reference genome enabled us to compare the QTLs from previous studies and to search for candidate genes. The markers generated here are valuable tools for introgressing specific root traits into elite germplasm. Importantly, the placement of markers onto the wheat reference genome enables more rigorous comparisons to be made between different mapping studies.

## 4. Materials and Methods

### 4.1. Germplasm

A population of hexaploid wheat RILs was generated using the wheat cultivars Maringa and Spica as parents. The Maringa genotype we used is a Brazilian cultivar that had the *Rht-B1b* semi-dwarfing gene [28] introgressed, while Spica is an older Australian cultivar with the wild-type *Rht* genes (tall) that has been used extensively in the study of late maturity α-amylase [29]. These genotypes differ significantly in various root traits including nodal root number, nodal root angle, seminal root angle and root hair length [10]. The Spica and Maringa parental lines first underwent single-seed descent for at least six generations before being crossed to generate an F_2_ population. Individual F_2_ seedlings were self-fertilised for six generations in a single-seed descent to generate RILs. The DNA extracted from the leaf tissue of these F_6:1_ seedlings was subjected to an Infinium wheat 90 K single nucleotide polymorphism (SNP) chip analysis which identified 14,047 polymorphic SNPs that were later used for QTL analysis [30]. These lines were then bulked to produce F_6:3_ RILs for phenotyping.

### 4.2. Screening

Plants were grown in tubes of soil in three growth cabinets (Conviron, Winnipeg, MB, Canada) with each line (227 RILs and 2 parental lines) replicated four times. Therefore, a total of 916 plants were assayed over 20 consecutive growth periods. The experiment was designed such that an average of 46 plants from a single cabinet were destructively sampled on each day of harvest. Using a design suitable for the best linear unbiased predictors (BLUP) analysis, four seeds of similar size for each of the 46 lines were placed on filter paper that had been moistened with deionized water that contained two drops of fungicide (1.4 g L^−1^ thiram). Lines were kept separate from one another by use of culture plates with 12 wells so that four seeds of each line occupied a single well. The seed was stratified at 4 °C for two days in the dark and then germinated by wrapping the culture plates with aluminium foil and incubating at room temperature for a further two days. Once germinated, one seedling from each of the lines was sown at a depth of 3 cm in a white polyvinylchloride tube (50 cm height and 8.6 cm inner diameter) that had been filled with 3.6 kg soil. The tubes were incubated in the growth cabinet (16 h day /8 h night, 23 °C/15 °C, 750 µmol m^−2^ s^−1^ light density) for 21 days before the plants were harvested.

The soil was prepared by mixing sieved potting soil with river sand (final composition 30% *v*/*v*) and 1 g L^−1^ Aboska fertiliser as described previously [10]. The soil was watered to pot capacity before sowing and then watered daily over the growth period. Each day a subsample of tubes from each cabinet was weighed to estimate the required volume of water to maintain soil moisture at pot capacity. At harvest, the plants and soil were gently removed from the pots, and the soil was washed off with tap water. Each plant was placed in a plastic bag with water for later screening. If there was insufficient time to complete the scoring on harvest day, the plants in plastic bags were stored overnight at 4 °C in the dark and scored the next day. The root traits assayed followed methods described previously [10]. The traits assayed included the root angle (the angle between the pair of second whorl leaf nodal roots on either side of the main stem, the pair of first whorl leaf nodal roots was measured if the second whorl had not yet developed); the total number of nodal roots present on the main stem and tillers; the nodal roots per stem (total nodal root number divided by the total number of stems); the root dry weight; and the average root diameter based on a WinRHIZO Pro (Regent Instruments Inc., Quebec, QC, Canada) analysis of scanned primary roots, root-to-shoot ratio and the specific root length (root length of scanned primary root divided by the dry weight of the primary root). Since some traits required values for shoot dry weight and the total number of stems (tillers plus main stem), QTL analyses were also performed for shoot dry weight and tiller number.

### 4.3. QTL Analysis

The genetic effect of BLUPs was derived for each dataset of the various traits (Supplementary Information Data Set1). The BLUP datasets of each trait were then searched for their QTL using the 90 K SNP data and the mpwgaim R package [31]. The above analyses were conducted in the R statistical computing environment [32], with the asreml package (version 3.1) for R used for all linear mixed model fits [33]. The wpwgaim R package provided genetic locations of flanking markers which were then converted to physical locations on the Chinese Spring reference genome, as described below.

### 4.4. Bioinformatics

To identify candidate genes, we first located the flanking markers for each QTL on the Chinese Spring reference genome (International Wheat Genome Sequencing Consortium (IWGSC) RefSeq v1.0; https://urgi.versailles.inra.fr accessed on 1 July 2022) using Pretzel Wheat (https://plantinformatics.io accessed on 1 July 2022). Pretzel Wheat is a web-based environment that enabled a Spica x Maringa genetic map (SNP data for the Spica and Maringa parents available at Pretzel Wheat) to be searched for physical locations on the Chinese Spring reference genome. If a marker was present on the Spica x Maringa genetic map but could not be found on the reference genome using Pretzel or directly via JBrowse at the Unité de Recherches en Génomique Info (URGI) website (https://urgi.versailles.inra.fr/jbrowseiwgsc/gmod_jbrowse accessed on 1 July 2022), we obtained the sequence of the SNP marker and undertook a basic local alignment search tool (BLAST) analysis against the wheat genome (https://urgi.versailles.inra.fr/blast/ accessed on 1 July 2022). Typically, BLAST generated three strong hits corresponding to the three genomes in wheat and we took the physical location for the marker that was located on the chromosome identified from the genetic map. If we were unable to obtain the sequence of the marker, we took the next closest flanking marker identified from the genetic map in Pretzel Wheat taking care to ensure the QTL’s physical size was over-estimated instead of under-estimated and used that marker’s physical location.

To determine if any previously cloned genes from monocotyledons could underlie the QTL identified in our study, we compiled a list of relevant genes from previous reports and located them on the wheat physical map. For genes that had been cloned from wheat, we located the gene as well as homeologs onto the physical map using BLAST analysis to identify the Chinese Spring gene that had an identical or near-identical sequence. For genes from other monocotyledonous species, we undertook a BLAST search at the URGI website using sequences of the coding regions as the query. Typically, BLAST generated three strong hits corresponding to the three genomes, and all three locations are shown on the list of physical locations. We used the same nomenclature used to describe the cloned genes except we preceded them with “Ta” to indicate *Triticum aestivum* and terminated them with the genome location of the homeolog (-A, -B or -D).

To establish if the QTL identified in the current study co-located with the QTL identified in previous studies, we used a published meta-analysis that identified candidate genes in hexaploid wheat that were underlying QTLs for a wide range of root traits. In that comprehensive study, Soriano and Alvaro [12] identified the candidate genes in hexaploid wheat that were underlying the QTL for a range of root traits from 30 studies using 31 different bi-parental populations. Candidate genes that were underlying the QTL used the IWSGC v1.0 nomenclature which enabled us to search JBrowse at the URGI website using gene names to identify physical locations. This allowed us to establish if any were located in or within 5 Mb of the QTL identified in our study (as defined by the flanking markers). Studies on wheat subsequent to 2019 that provided physical locations for the QTL of root traits were also included in the search for overlapping QTL [13,14,34,35].

## Figures and Tables

**Table 1:** The heritability of the various traits.

Trait	Heritability
Root dry weight	0.71
Root diameter	0.71
Total nodal root number	0.80
Nodal root number per stem	0.80
Root angle	0.33
Specific root length	0.63
Root-to-shoot ratio	0.62
Shoot dry weight	0.80
Tiller number	0.77

**Table 2:** Summary of QTLs showing the number of QTLs with LOD > 3.0, the number of QTLs with LOD < 3.0 and the per cent of the variation explained by summing all the QTLs for each trait.

Trait	Total # of QTLs	# of QTLs with LOD > 3.0	# of QTLs with LOD < 3.0	% Variation Explained
**Root dry weight**	12	3	9	54.9
**Root diameter**	10	7	3	72.5
**Total nodal root number**	6	5	1	43.9
**Nodal root number per stem**	8	5	3	54.4
**Root angle**	5	1	4	66.4
**Specific root length**	7	2	5	52.7
**Root-to-shoot ratio**	6	1	5	44.6
**Shoot dry weight**	9	3	6	46.2
**Tiller number**	4	0	4	22.7

**Table 3:** Physical marker locations of root QTLs with a LOD > 3.0. Chromosome locations, flanking marker positions and intervals of QTLs are shown for the reference Chinese Spring genome.

QTL: Name, Trait, and Chromosome	Left Marker	Physical Location Mb	Right Marker	Physical Location Mb	Interval Mb
**Root dry weight**					
*Qrdw-2A*	wsnp_Ra_c66636_64922359 *	740.37	RAC875_c66695_1376	743.39	3.02
*Qrdw-2B*	Excalibur_c34380_275	213.89	Excalibur_c30207_261 *	412.71	198.82
*Qrdw-4A*	Excalibur_c539_1253	597.69	wsnp_Ku_c1205_2398925	597.91	0.22
**Root diameter**					
*Qrd-4A*	BS00109722_51	544.62	wsnp_Ex_c1373_2628597	555.58	10.96
*Qrd-4B1*	RAC875_c56905_265	42.54	BS00063035_51	43.18	0.64
*Qrd-4B2*	tplb0036n21_1765	506.14	IAAV9209 *	507.98	1.84
*Qrd-5A*	wsnp_Ex_c57094_58953404 *	9.66	RAC875_c30001_200	10.04	0.38
*Qrd-5D*	RAC875_c51455_182	79.99	BS00021901_51	192.27	112.28
*Qrd-6A*	Tdurum_contig30082_197	109.38	Excalibur_c49419_202	127.19	17.81
*Qrd-7A*	IAAV1372	131.86	BS00073170_51	133.67	1.81
**Total nodal root number**					
*Qtnrn-2B*	RAC875_c102981_396	249.37	Kukri_c691_504	384.67	135.3
*Qtnrn-4B*	Tdurum_contig30537_228	18.42	Tdurum_contig33737_157	37.69	19.27
*Qtnrn-6B*	JD_c8399_626 *	41.70	Tdurum_contig55201_928	69.55	27.85
*Qtnrn-7A1*	D_GDEEGVY01CQJ66_272 *	114.28	Tdurum_contig63598_188	115.81	1.53
*Qtnrn-7A2*	Excalibur_c12500_116	71.67	BS00063555_51 **	78.22	6.55
**Nodal root number per stem**					
*Qnrns-2B*	TA006140-0798	560.89	Ku_c66850_510	579.57	18.68
*Qnrns-4B*	Jagger_c1432_289	480.92	Tdurum_contig11735_1294	487.77	6.85
*Qnrns-6D*	D_contig00170_262	244.01	Kukri_c35951_337 **	258.36	14.35
*Qnrns-7A*	Excalibur_c12500_116	71.67	BS00063555_51 **	78.22	6.55
*Qnrns-7B*	BS00055861_51	558.32	RAC875_c79695_343 *	559.71	1.39
**Root angle**					
*Qra-4B*	Jagger_c1432_289	480.92	Tdurum_contig11735_1294	487.77	6.85
**Specific root length**					
*Qsrl-4B1*	tplb0036n21_1765	506.14	Tdurum_contig64848_104	518.68	12.54
*Qsrl-6A*	CAP12_c2701_221	559.53	Tdurum_contig28847_322	563.13	3.6
**Root-to-shoot ratio**					
*Qrsr-3A*	IACX333	674.75	BS00050109_51	680.75	6.00
**Shoot dry weight**					
*Qsdw-2A*	Kukri_c18104_1416	776.22	BobWhite_c16248_382	779.82	3.60
*Qsdw-3B*	RAC875_c48556_278	610.18	RAC875_c28912_306	610.75	0.57
*Qsdw-4A*	wsnp_Ex_c7011_12080274	146.56	IACX1427	381.22	234.66

* A BLAST of the marker sequence was used to identify the physical location. ** Location of the flanking marker was not available, so the next closest marker was identified.

**Table 4:** Physical marker locations of root QTLs with a LOD < 3.0. Chromosome locations, flanking marker positions, and intervals of QTLs are shown for the reference Chinese Spring genome.

QTL: Name, Trait and Chromosome	Left Marker	Physical Location Mb	Right Marker	Physical Location Mb	Interval Mb
**Root dry weight**					
*Qrdw-1A*	RAC875_c1599_342	575.36	BS00022824_51	577.47	2.11
*Qrdw-1B*	CAP7_c3847_204	17.62	Excalibur_rep_c107678_98	19.02	1.4
*Qrdw-4B*	BS00011085_51 *	407.41	TA004394-0527	423.36	15.95
*Qrdw-5A*	BS00088851_51	591.46	RAC875_rep_c76193_513	591.49	0.03
*Qrdw-5B*	Tdurum_contig28552_211	707.13	BS00021993_51	712.90	5.77
*Qrdw-6B*	BobWhite_c42198_254	471.16	Excalibur_c2328_1207	472.61	1.45
*Qrdw-6D1*	BobWhite_c34996_280 *	460.70	wsnp_Ex_c14691_22763171 *	461.36	0.66
*Qrdw-6D2*	D_contig00170_262 **	244.01	RFL_Contig6056_604	290.82	46.81
*Qrdw-7A*	Tdurum_contig8615_370	657.02	RAC875_c35723_106 *	657.72	0.70
**Root diameter**					
*Qrd-3A*	GENE-4795_75	556.46	wsnp_Ex_rep_c66685_65003254	571.41	14.95
*Qrd-5B*	wsnp_Ra_c24619_34168104 *	508.80	BS00068200_51 *	512.25	3.45
*Qrd-7B*	Excalibur_c13444_235	613.18	RAC875_rep_c73965_114	616.41	3.23
**Total nodal root number**					
*Qtnrn-3B*	BS00049639_51	419.57	wsnp_JD_c10233_10936535 *	424.79	5.22
**Nodal root number per stem**					
*Qnrns-2A1*	Tdurum_contig66353_358	758.31	RAC875_rep_c83950_222 *	759.84	1.53
*Qnrns-2A2*	RAC875_c510_923 **	5.91	Excalibur_c12980_2621	7.55	1.64
*Qnrns-3A*	IACX333	674.75	BS00050109_51	680.75	6.00
**Root angle**					
*Qra-1A*	D_contig04348_649 *	365.54	GENE-0235_245	381.32	15.78
*Qra-3A*	wsnp_CAP8_c6939_3242530	714.16	Kukri_rep_c106620_208	714.30	0.14
*Qra-5B*	wsnp_Ku_c11138_18252461	659.97	GENE-2582_259 **	662.04	2.07
*Qra-6B*	Excalibur_c13206_108	697.54	CAP11_c816_470*	701.66	4.12
**Specific root length**					
*Qsrl-3B*	Tdurum_contig45817_193	771.13	Kukri_c43588_354	771.94	0.81
*Qsrl-4A*	BS00109722_51	544.62	wsnp_Ex_c1373_2628597 *	555.58	10.96
*Qsrl-4B2*	BS00063035_51	43.18	Excalibur_c56787_95	59.21	16.03
*Qsrl-5D*	BS00021901_51	192.27	Kukri_c13045_302	241.25	48.98
*Qsrl-7A*	GENE-4508_109	436.79	BobWhite_c5396_296	442.27	5.48
**Root to shoot ratio**					
*Qrsr-2A*	BS00012320_51	647.93	BS00081195_51	676.23	28.30
*Qrsr-3B*	BS00022051_51	545.01	RFL_Contig4667_3535 *	545.65	0.64
*Qrsr-3D*	RFL_Contig2471_119 **	17.41	Kukri_c908_584	22.37	4.96
*Qrsr-7B*	Tdurum_contig74753_946	601.23	Kukri_c9405_379	607.58	6.35
*Qrsr-7D*	D_GBQ4KXB02FR7XF_153	632.34	RAC875_c59686_292 *	633.01	0.67
**Shoot dry weight**					
*Qsdw-6D*	wsnp_BE445201D_Ta_1_1	105.54	RFL_Contig6056_604	290.82	185.28
**Tiller number**					
*Qtn-3A*	BS00084158_51	671.14	IACX333	674.75	3.61
*Qtn-4B*	tplb0024a16_411 *	15.42	wsnp_Ex_c6739_11646407 *	17.26	1.84
*Qtn-6A1*	Excalibur_c96749_512	609.56	Kukri_c40994_61	609.97	0.41
*Qtn-6A2*	Tdurum_contig30082_197	109.38	Excalibur_c49419_202	127.19	17.81

* A BLAST of the marker sequence was used to identify the physical location. ** Location of the flanking marker was not available, so the next closest marker was identified.

## Data Availability

The datasets generated during and/or analysed during the current study are available from the corresponding author upon reasonable request.

## Supplementary Materials

The following supplementary information are available online at https://www.mdpi.com/article/10.3390/ijms241310492/s1. Refs. [36,37,38,39,40,41,42,43,44,45,46,47,48,49,50,51,52,53,54,55,56,57,58,59,60,61,62,63,64,65] are cited in Supplementary Materials.
