# Supplementary material for: Physical Mapping of QTLs for Root Traits in a Population of Recombinant Inbred Lines of Hexaploid Wheat
Source: Int J Mol Sci. 2023 Jun 22;24(13):10492. doi: 10.3390/ijms241310492 (PMC10341896; doi:10.3390/ijms241310492)
Supplement: Supplementary file 1 [file ijms-24-10492-s001.zip › Supplementary Tables S1-S3.pdf]

Supplementary Information Tables S1–S3.

Physical mapping of QTLs for root traits in a population of recombinant inbred lines of hexaploid wheat

Xiaoqing Li [1,*], Anton P. Wasson [1], Alexander B. Zwart [1], Alex Whan [1], Peter R. Ryan [1], Kerrie Forrest [2], Matthew Hayden [2,3], Sabrina Chin [4], Richard Richards [1] and Emmanuel Delhaize [5]

[1] CSIRO Agriculture and Food, Canberra, ACT 2601, Australia; anton.wasson@csiro.au (A.P.W.); alec.zwart@data61.csiro.au (A.B.Z.); alex.whan@csiro.au (A.W.); peter.ryan@csiro.au (P.R.R.); richard.richards@csiro.au (R.R.)
[2] Agriculture Victoria Research, AgriBio, Centre for AgriBioscience, Bundoora, VIC 3083, Australia; kerrie.forrest@agriculture.vic.gov.au (K.F.); matthew.hayden@agriculture.vic.gov.au (M.H.)
[3] School of Applied Systems Biology, La Trobe University, Bundoora, VIC 3086, Australia
[4] Department of Botany, University of Wisconsin, Madison, WI 53706, USA; schin7@wisc.edu
[5] Australian Plant Phenomics Facility, Research School of Biology, The Australian National University, Canberra, ACT 2601, Australia; manny.delhaize@anu.edu.au
* Correspondence: xiaoqing.li@csiro.au

Table S1. Genetic map of root QTL with a LOD > 3.0. Shown are flanking marker positions in cM, LOD score, additive effect and percent variation contributed by the QTL.

QTL: name, trait and chromosome	Left marker	cM	Right marker	cM	LOD	Add*	% var'n
**Root dry weight**							
Qrdw-2A	wsnp_Ra_c66636_64922359	183.8	RAC875_c66695_1376	185.2	3.91	-0.013	7.0
Qrdw-2B	Excalibur_c30207_261	157.0	Excalibur_c34380_275	159.18	5.29	0.017	10.6
Qrdw-4A	Excalibur_c539_1253	138.9	wsnp_Ku_c1205_2398925	141.1	3.54	0.013	6.4
**Root diameter**							
Qrd-4A	BS00109722_51	104.2	wsnp_Ex_c1373_2628597	106.1	4.08	0.003	5.9
Qrd-4B1	RAC875_c56905_265	85.3	BS00063035_51	87.2	3.10	0.004	7.4
Qrd-4B2	IAAV9209	135.8	tplb0036n21_1765	137.3	6.82	-0.006	15.6
Qrd-5A	wsnp_Ex_c57094_58953404	24.8	RAC875_c30001_200	27.4	3.92	-0.003	4.9
Qrd-5D	RAC875_c51455_182	32.3	BS00021901_51	33.7	11.44	-0.005	13.8
Qrd-6A	Tdurum_contig30082_197	106.4	Excalibur_c49419_202	108.2	5.90	-0.004	7.6
Qrd-7A	IAAV1372	184.7	BS00073170_51	186.2	5.26	-0.004	7.5
**Total nodal root number**							
Qnrn-2B	RAC875_c102981_396	141.6	Kukri_c691_504	143.2	3.16	0.858	6.0
Qnrn-4B	Tdurum_contig33737_157	77.8	Tdurum_contig30537_228	79.6	4.28	-1.014	8.2
Qnrn-6B	JD_c8399_626	69.5	Tdurum_contig55201_928	81.7	3.69	-0.979	6.3
Qnrn-7A1	D_GDEEGVY01CQJ66_272	165.2	Tdurum_contig63598_188	167.2	3.13	-0.902	6.5
Qnrn-7A2	Excalibur_c12500_116	123.6	Tdurum_contig82510_574	126.9	6.28	-1.269	12.2
**Nodal root number per stem**							
Qnrns-2B	TA006140-0798	181.8	Ku_c66850_510	184.5	3.86	0.088	6.0
Qnrns-4B	Jagger_c1432_289	129.7	Tdurum_contig11735_1294	131.6	4.52	-0.096	7.2
Qnrns-6D	RFL_Contig6056_604	2.9	CAP7_c2537_344	4.6	3.48	0.080	5.1
Qnrns-7A	Excalibur_c12500_116	123.6	Tdurum_contig82510_574	126.9	13.14	-0.167	20.2
Qnrns-7B	BS00055861_51	133.9	RAC875_c79695_343	135.9	3.01	0.078	4.8
**Root angle**							
Qra-4B	Jagger_c1432_289	129.7	Tdurum_contig11735_1294	131.6	5.01	-0.026	24.1
**Specific root length**							
Qsrl-4B1	tplb0036n21_1765	137.3	Tdurum_contig64848_104	138.6	5.50	0.017	19.6
Qsrl-6A	CAP12_c2701_221	135.2	Tdurum_contig28847_322	138.1	4.62	0.012	9.5
**Root to shoot ratio**							
Qrsr-3A	IACX333	170.2	BS00050109_51	172.0	5.92	-0.008	15
**Shoot dry weight**							
Qsdw-2A	BobWhite_c16248_382	209.1	Kukri_c18104_1416	210.7	3.32	-0.04	5.5
Qsdw-3B	RAC875_c48556_278	183.6	RAC875_c28912_306	185.4	6.51	-0.059	11.2
Qsdw-4A	wsnp_Ex_c7011_12080274	94.0	IACX1427	95.4	3.11	0.035	5.9

*Positive alleles contributed by Spica and negative alleles contributed by Maringa.

Table S2. Genetic map of root QTL with a LOD < 3.0. Shown are flanking marker positions in cM, LOD score, additive effect and percent variation contributed by the QTL.

QTL: name, trait and chromosome	Left marker	cM	Right marker	cM	LOD	Add*	% var'n
**Root dry weight**							
Qrdw-1A	RAC875_c1599_342	260.9	BS00022824_51	266.1	2.722	-0.011	4.5
Qrdw-1B	Excalibur_rep_c107678_98	287.8	CAP7_c3847_204	290.1	1.654	-0.008	3.0
Qrdw-4B	TA004394-0527	126.2	BS00011085_51	128.2	1.613	0.009	3.0
Qrdw-5A	RAC875_rep_c76193_513	125.9	BS00088851_51	128.0	2.764	0.011	4.8
Qrdw-5B	BS00021993_51	0.0	Tdurum_contig28552_211	8.7	1.967	0.010	3.3
Qrdw-6B	Excalibur_c2328_1207	165.8	BobWhite_c42198_254	166.6	1.378	-0.008	2.6
Qrdw-6D1	wsnp_Ex_c14691_22763171	27.4	BobWhite_c34996_280	28.3	2.198	0.009	3.6
Qrdw-6D2	CAP7_c2537_344	114.8	RFL_Contig6056_604	116.5	1.36	0.007	2.2
Qrdw-7A	RAC875_c35723_106	110.7	Tdurum_contig8615_370	112.6	2.144	-0.010	3.9
**Root diameter**							
Qrd-3A	wsnp_Ex_rep_c66685_65003254	178.8	GENE-4795_75	180.2	2.70	-0.003	3.5
Qrd-5B	BS00068200_51	229.9	wsnp_Ra_c24619_34168104	234.7	2.93	0.003	3.7
Qrd-7B	RAC875_rep_c73965_114	116.8	Excalibur_c13444_235	118.8	2.00	-0.002	2.6
**Total nodal root number**							
Qnrn-3B	wsnp_JD_c10233_10936535	212.6	BS00049639_51	214.6	2.514	-0.763	4.8
**Nodal root number per stem**							
Qnrns-2A1	RAC875_rep_c83950_222	16.9	Tdurum_contig66353_358	18.5	2.459	-0.067	3.6
Qnrns-2A2	Kukri_rep_c109732_72	215.8	Excalibur_c12980_2621	217.2	2.693	0.070	3.9
Qnrns-3A	BS00050109_51	112.6	IACX333	114.4	2.348	-0.066	3.6
**Root angle**							
Qra-1A	GENE-0235_245	104.4	D_contig04348_649	107.5	2.329	-0.017	10.9
Qra-3A	Kukri_rep_c106620_208	50.7	wsnp_CAP8_c6939_3242530	51.6	2.558	-0.017	11.1
Qra-5B	RAC875_c31261_367	101.0	wsnp_Ku_c11138_18252461	101.6	2.248	-0.016	10.2
Qra-6B	CAP11_c816_470	46.7	Excalibur_c13206_108	50.5	2.292	-0.017	10.1
**Specific root length**							
Qsrl-3B	Kukri_c43588_354	62.9	Tdurum_contig45817_193	64.7	1.616	-0.007	3.2
Qsrl-4A	wsnp_Ex_c1373_2628597	195.4	BS00109722_51	197.3	1.803	-0.007	3.8
Qsrl-4B2	Excalibur_c56787_95	152.4	BS00063035_51	154.7	1.714	-0.009	6.5
Qsrl-5D	Kukri_c13045_302	203.0	BS00021901_51	204.8	2.255	0.008	4.4
Qsrl-7A	BobWhite_c5396_296	203.5	GENE-4508_109	205.5	2.628	0.009	5.7
**Root to shoot ratio**							
Qrsr-2A	BS00081195_51	104.4	BS00012320_51	109.4	1.886	-0.005	5.0
Qrsr-3B	RFL_Contig4667_3535	187.8	BS00022051_51	189.1	2.157	0.005	5.8
Qrsr-3D	Kukri_c908_584	183.2	BobWhite_c27145_731	184.3	2.197	0.005	5.4
Qrsr-7B	Kukri_c9405_379	128.4	Tdurum_contig74753_946	130.8	2.73	0.006	6.9
Qrsr-7D	D_GBQ4KXB02FR7XF_153	0.0	RAC875_c59686_292	1.3	2.824	0.005	6.5
**Shoot dry weight**							
Qsdw-6D	RFL_Contig6056_604	116.5	wsnp_BE445201D_Ta_1_1	119.4	1.87	0.029	2.9
**Tiller number**							
Qtn-3A	IACX333	114.4	BS00084158_51	117.3	2.896	0.338	6.9
Qtn-4B	wsnp_Ex_c6739_11646407	200.5	tplb0024a16_411	203.1	1.915	-0.266	4.5
Qtn-6A1	Excalibur_c96749_512	22.0	Kukri_c40994_61	22.4	2.957	-0.331	6.8
Qtn-6A2	Excalibur_c49419_202	145.6	Tdurum_contig30082_197	147.4	1.941	0.267	4.5

*Positive alleles contributed by Spica and negative alleles contributed by Maringa.

Table S3. List of wheat root genes and the wheat homologs of genes associated with root development and architecture cloned from other monocotyledons. Gene names used the same nomenclature as original references and are preceded with *Ta* to indicate *Triticum aestivum*, followed by designation of the particular homeolog (*-A*, *-B* or *-D*). Gene names and physical locations are provided for the IWGSC v1.0 genome. A brief description of the trait, reference, and species from which the genes were cloned are also provided.

Gene name	Trait	Species	Reference	IWGSC v 1.0 gene name	Physical location
*TaMOR-A**	Root architecture	Wheat	[36]	*TraesCS4A02G415400*	chr4A: 685380302-685381598
TaMOR-B				*TraesCS4B02G316200*	chr4B: 605691920-605693239
TaMOR-D				*TraesCS4D02G312800*	chr4D: 478997945-478999338
TaANDRO-A	Deeper rooting	Wheat	[37]	*TraesCS5A02G213300*	chr5A: 428994186-428997632
TaBNDRO-B				*TraesCS5B02G210500*	chr5B: 381041995-381044714
TaDNDRO-D				*TraesCS5D02G218700*	chr5D: 327631371-327634216
TaARF4-A	Root and shoot traits-auxin response factors	Wheat	[38]	*TraesCS3A02G442000*	chr3A: 684040773-684045606
TaARF4-B				*TraesCS3B02G475800*	chr3B: 724737433-724742425
TaARF4-D				*TraesCS3D02G434700*	chr3D: 547298032-547302973
TaSERK-A	Root and shoot traits	Wheat	[39]	*TraesCS2A02G343100*	chr2A: 580017609-580023752
TaSERK-B				*TraesCS2B02G340700*	chr2B: 486190984-486197028
TaSERK-D				*TraesCS2D02G321400*	chr2D: 412833158-412839366
TaBRI1-A	Interacts with *TaSERK*	Wheat	[40]	*TraesCS3A02G245000*	chr3A: 458678631-458682691
TaBRI1-B				*TraesCS3B02G275000*	chr3B: 443803664-443807278
TaBRI1-D				*TraesCS3D02G246500*	chr3D: 344337925-344342369
TaVP1-A	Vacuolar-PPase	Wheat	[41]	*TraesCS7A02G141300*	chr7A: 92888389-92893250
TaVP1-B				*TraesCS7B02G042600*	chr7B: 42027289-42032517
TaVP1-D				*TraesCS7D02G142600*	chr7D: 90848363-90854069
TaVRN-A1	Root system architecture	Wheat	[18]	*TraesCS5A02G391700*	chr5A: 587411454-587423416
TaVRN-B1				*TraesCS5B02G396600*	chr5B: 573802883-573816070
TaVRN-D1				*TraesCS5D02G401500*	chr5D: 467176609-467184508
TaPSTOL	Root biomass	Wheat	[42]	Not annotated	chr5A: 41374604-41378275
**Other species**					
TaCRL5-A	Crown roots	Rice *CRL5*	[17]	*TraesCS2A02G267600*	chr2A: 428908059-428911931
TaCRL5-B				*TraesCS2B02G268100*	chr2B: 361422652-361426297
TaCRL5-D				*TraesCS2D02G256600*	chr2D: 311715447-311719061
TaEXP10-A	Root cell elongation	Rice Os*EXP10*	[19]	*TraesCS2A02G393700*	chr2A: 643439927-643444151
TaEXP10-B				*TraesCS2B02G411700*	chr2B: 585891499-585895732
TaEXP10-D				*TraesCS2D02G391600*	chr2D: 498768162-498772341
TaSNAC1-A	Root size	Rice *SNAC1*	[43]	*TraesCS5A02G468300*	chr5A: 645726140-645727775
TaSNAC1-B				*TraesCS5B02G480900*	chr5B: 653432387-653434033
TaSNAC1-D				*TraesCS5D02G481200*	chr5D:519277079-519278774
TaSOR1-A	Root ethylene responses	Rice *SOR1*	[44]	*TraesCS2A02G045100*	chr2A: 17738033-17741923
TaSOR1-B				*TraesCS2B02G057300*	chr2B: 27895909-27899621
TaSOR1-D				*TraesCS2D02G043700*	chr2D: 15730614-15734542
TaRUM1-A	Root initiation	Maize *RUM1*	[45]	*TraesCS3A02G270000*	chr3A: 496704577-496707472
TaRUM1-B				*TraesCS3B02G303900*	chr3B: 487906086-487911438
TaRUM1-D				*TraesCS3D02G269700*	chr3D: 374205888-374211351
TaPIN1-A	Root emergence and tillering	Rice *OsPIN1*	[46]	*TraesCS4A02G188100*	chr4A: 466938697-466941682
TaPIN1-B				*TraesCS4B02G130100*	chr4B: 170926810-170929710
TaPIN1-D				*TraesCS4D02G125300*	chr4D: 109469533-109472292

TaWOX11-A	Crown root development	Rice *WOX11*	[47]	*TraesCS2A02G100700*	chr2A: 53782606-53785288
TaWOX11-B				*TraesCS2B02G117900*	chr2B: 81755546-81758516
TaWOX11-D				*TraesCS2D02G100200*	chr2D: 52227203-52229885
TaGNA1-A	Root cell shape	Rice *OsGNA1*	[48]	*TraesCS5A02G264900*	chr5A: 476850991-476855275
TaGNA1-B				*TraesCS5B02G264500*	chr5B: 448600463-448601077
TaGNA1-D				*TraesCS5D02G272900*	chr5D: 375745767-375748324
TaSPR1-A	Short postembryoni c root	Rice *OsSPR1*	[49]	*TraesCS3A02G397500*	chr3A: 644803705-644811301
TaSPR1-B				*TraesCS3B02G429400*	chr3B: 668069692-668076933
TaSPR1-D				*TraesCS3D02G391300*	chr3D: 506172242-506178602
TaCOW1-A	Root to shoot ratio	Rice *OsCOW1*	[50]	*TraesCS4A02G027500*	chr4A: 19447812-19450783
TaCOW1-B				*TraesCS4B02G278300*	chr4B: 560663067-560665760
TaCOW1-D				*TraesCS4D02G276600*	chr4D: 447910002-447913028
TaWAK1-A1	Root growth	*Barley HvWAK1*	[51]	*TraesCS4A02G499928*	chr4A: 744536044-744539773
TaWAK1-A2				*TraesCS7A02G000100*	chr7A: 238043-241215
TaHDAC1-A	Lateral root formation	Rice *OsHDAC1*	[52]	*TraesCS7A02G365600*	chr7A: 539543850-539554190
TaHDAC1-B				*TraesCS7B02G261800*	chr7B: 482237779-482248552
TaHDAC1-D				*TraesCS7D02G356800*	chr7D: 459923113-459936250
TaGSK2-A	Lateral root formation	Rice *OsGSK2*	[52]	*TraesCS3A02G136500*	chr3A: 114041077-114045106
TaGSK2-D				*TraesCS3D02G137200*	chr3D: 95945752-95950119
TaBZR1-A	Lateral root formation	Rice *OsBZR1*	[52]	*TraesCS2A02G187800*	chr2A: 150123158-150124982
TaBZR1-B				*TraesCS2B02G219300*	chr2B: 209436541-209438293
TaBZR1-D				*TraesCS2D02G199900*	chr2D: 151325675-151327278
TaCRD1-A	Crown root development	Rice *CRD1*	[53]	*TraesCS3A02G199400*	chr3A: 325281188-325311105
TaCRD1-B				*TraesCS3B02G229100*	chr3B: 335836563-335873333
TaCRD1-D				*TraesCS3D02G205700*	chr3D: 264696256-264720565
TaEGT1-A	Root angle	Barley *EGT1*	[54]	*TraesCS6A02G272800*	chr6A: 499397353-499400701
TaEGT1-B				*TraesCS6B02G300200*	chr6B: 538363931-538367345
TaEGT1-D				*TraesCS6D02G252800*	chr6D: 357002985-357006388
TaEGT2-A	Root angle	Barley *EGT2*	[20]	*TraesCS5A02G102000*	chr5A: 151732800-151736140
TaEGT2-B				*TraesCS5B02G164200*	chr5B: 143293893-143296491
TaEGT2-D				*TraesCS5D02G171500*	chr5D: 131504758-131508027
TaIAA11-A	Lateral root growth	Rice *OsIAA11*	[55]	*TraesCS5A02G378300*	chr5A: 575657011-575659289
TaIAA11-B				*TraesCS5B02G381900*	chr5B: 559990916-559992903
TaIAA11-D				*TraesCS5D02G388300*	chr5D: 457775681-457777965
TaIAA13-A	Lateral root growth	Rice *OsIAA13***	[56]	*TraesCS5A02G382600*	chr5A: 580464947-580467685
TaIAA13-B				*TraesCS5B02G386800*	chr5B:565928821-565931697
TaIAA13-D				*TraesCS5D02G392000*	chr5D:460393490-460396369
TaIAA9-A	Gravitropic response	Rice *OsIAA9*	[57]	*TraesCS6A02G373300*	chr6A: 596739621-596740759
TaIAA9-B				*TraesCS6B02G411000*	chr6B: 685017015-685018041
TaIAA9-D				*TraesCS6D02G357500*	chr6D: 451082161-451083072
*TaIAA15a-A****	Gravitropic response	Rice *OsIAA15*	[16]	*TraesCS7A02G371500*	chr7A: 545472957-545474340
*TaIAA15a-B****				*TraesCS7B02G256100*	chr7B: 474642958-474643848
*TaIAA15a-D****				*TraesCS7D02G351400*	chr7D: 452153857-452154744
TaIAA3-A	Gravitropic response	Rice *OsIAA3*	[58]	*TraesCS5A02G058700*	chr5A: 57562924-57564218
TaIAA3-B				*TraesCS5B02G058800*	chr5B: 64418744-64420036
TaIAA3-D				*TraesCS5D02G069200*	chr5D: 66667985-66669320
TaPIN2-A	Root angle	Rice *OsPIN2*	[59]	*TraesCS7A02G492400*	chr7A: 681011336-681015619
TaPIN2-B				*TraesCS7B02G398100*	chr7B: 664106649-664110679
TaPIN2-D				*TraesCS7D02G478800*	chr7D: 589512605-589516322
TaCYP2-A	Lateral root development	Rice *OsCYP2*	[60]	*TraesCS6A02G068900*	chr6A: 37407147-37408119

TaCYP2-B				*TraesCS6B02G093100*	chr6B: 68922518-68923420
TaCYP2-D				*TraesCS6D02G066700*	chr6D: 32693962-32694930
TaRSA3.2-A	Root angle	Maize *ZmRSA3.2*	[61]	*TraesCS3A02G397900*	chr3A: 645129450-645133585
TaRSA3.2-B				*TraesCS3B02G429900*	chr3B :669424620-669428960
TaRSA3.2-D				*TraesCS3D02G391800*	chr3D: 507026845-507030741
TaCBLI-A	Root angle	Maize *CBLI*	[62]	*TraesCS5A02G377900*	chr5A: 575594327-575595945
TaCBLI-B				*TraesCS5B02G381500*	chr5B: 559763470-559765063
TaCBLI-D				*TraesCS5D02G387900*	chr5D :457663678-457665461
TaBIGE-A	Seminal root number	Maize *BIGE*	[63]	*TraesCS4A02G350200*	chr4A: 626439275-626443664
TaBIGE-B				*TraesCS5B02G522900*	chr5B: 684227667-684231381
TaBIGE-D				*TraesCS5D02G521600*	chr5D :542589766-542592882

* The *TaMOR* genes of wheat are homologous to the *CRL1* and *RTCS* genes originally identified in rice and maize [64].

** The recently cloned gene *ZmRSA3.1* from maize that controls root angle is a homologue of *OsIAA13* [61].

*** A *TaIAA15* gene was previously described as controlling shoot architecture [65]. To differentiate the wheat homologs of *OsIAA15* from *TaIAA15* we included the suffix "a" after 15 in the gene name.

Supplementary References

16. Yan DW, Wang J, Yuan TT, Hong LW, Gao X, Lu YT (2013) Perturbation of auxin homeostasis by overexpression of wild-type *IAA15* results in impaired stem cell differentiation and gravitropism in roots. PLoS One 8:e58103. doi: 10.1371/journal.pone.0058103
17. Kitomi Y, Ito H, Hobo T, Aya K, Kitano H, Inukai Y (2011) The auxin responsive AP2/ERF transcription factor *CROWN ROOTLESS5* is involved in crown root initiation in rice through the induction of *OsRR1*, a type-A response regulator of cytokinin signaling. Plant J 67:472-484. doi: 10.1111/j.1365-313X.2011.04610.x
18. Voss-Fels KP, Robinson H, Mudge SR, Richard C, Newman S, Wittkop B, Stahl A, Friedt W, Frisch M, Gabur I, Miller-Cooper A, Campbell BC, Kelly A, Fox G, Christopher J, Christopher M, Chenu K, Franckowiak J, Mace ES, Borrell AK, Eagles H, Jordan DR, Botella JR, Hammer G, Godwin ID, Trevaskis B, Snowdon RJ, Hickey LT (2018) *VERNALIZATION1* modulates root system architecture in wheat and barley. Mol Plant 11:226-229. doi: 10.1016/j.molp.2017.10.005
19. Che J, Yamaji N, Shen RF, Ma JF (2016) An Al-inducible expansin gene, *OsEXPA10* is involved in root cell elongation of rice. Plant J 88:132-142. doi: 10.1111/tpj.13237
20. Kirschner GK, Rosignoli S, Guo L, Vardanega I, Imani J, Altmuller J, Milner SG, Balzano R, Nagel KA, Pflugfelder D, Forestan C, Bovina R, Koller R, Stocker TG, Mascher M, Simmonds J, Uauy C, Schoof H, Tuberosa R, Salvi S, Hochholdinger F (2021) *ENHANCED GRAVITROPISM 2* encodes a STERILE ALPHA MOTIF-containing protein that controls root growth angle in barley and wheat. Proc Natl Acad Sci USA 118:e2101526118. doi: 10.1073/pnas.2101526118
36. Li C, Wang J, Li L, Li J, Zhuang M, Li B, Li Q, Huang J, Du Y, Wang J, Fan Z, Mao X, Jing R (2022a) *TaMOR* is essential for root initiation and improvement of root system architecture in wheat. Plant Biotechnol J 20:862-875. doi: 10.1111/pbi.13765
37. Ashraf A, Rehman OU, Muzammil S, Leon J, Naz AA, Rasool F, Ali GM, Zafar Y, Khan MR (2019) Evolution of *Deeper Rooting 1-like* homoeologs in wheat entails the C-

terminus mutations as well as gain and loss of auxin response elements. PLoS One 14:e0214145. doi: 10.1371/journal.pone.0214145

38. Wang J, Wang R, Mao X, Li L, Chang X, Zhang X, Jing R (2019) *TaARF4* genes are linked to root growth and plant height in wheat. Ann Bot 124:903-915. doi: 10.1093/aob/mcy218
39. Singh A, Khurana P (2017) Ectopic expression of *Triticum aestivum SERK* genes (*TaSERKs*) control plant growth and development in *Arabidopsis*. Sci Rep 7:12368. doi: 10.1038/s41598-017-10038-1
40. Singh A, Breja P, Khurana JP, Khurana P (2016) Wheat *Brassinosteroid-Insensitive1* (*TaBRI1*) interacts with members of *TaSERK* gene family and cause early flowering and seed yield enhancement in *Arabidopsis*. PLoS ONE 11(6): e0153273. doi:10.1371/journal.pone.0153273

41. Wang Y, Xu H, Zhang G, Zhu H, Zhang L, Zhang Z, Zhang C, Ma Z (2009) Expression and responses to dehydration and salinity stresses of V-PPase gene members in wheat. J Genet Genomics 36:711-720. doi: 10.1016/S1673-8527(08)60164-2
42. Milner MJ, Howells RM, Craze M, Bowden S, Graham N, Wallington EJ (2018) A PSTOL-like gene, *TaPSTOL*, controls a number of agronomically important traits in wheat. BMC Plant Biol 18:115. doi: 10.1186/s12870-018-1331-4
43. Liu G, Li X, Jin S, Liu X, Zhu L, Nie Y, Zhang X (2014) Overexpression of rice NAC gene *SNAC1* improves drought and salt tolerance by enhancing root development and reducing transpiration rate in transgenic cotton. PLoS One 9:e86895. doi: 10.1371/journal.pone.0086895
44. Chen H, Ma B, Zhou Y, He SJ, Tang SY, Lu X, Xie Q, Chen SY, Zhang JS (2018) E3 ubiquitin ligase SOR1 regulates ethylene response in rice root by modulating stability of Aux/IAA protein. Proc Natl Acad Sci USA 115:4513-4518. doi: 10.1073/pnas.1719387115
45. von Behrens I, Komatsu M, Zhang Y, Berendzen KW, Niu X, Sakai H, Taramino G, Hochholdinger F (2011) *Rootless with undetectable meristem 1* encodes a monocot-specific AUX/IAA protein that controls embryonic seminal and post-embryonic lateral root initiation in maize. Plant J 66:341-353. doi: 10.1111/j.1365-313X.2011.04495.x
46. Xu M, Zhu L, Shou H, Wu P (2005) A *PIN1* family gene, *OsPIN1*, involved in auxin-dependent adventitious root emergence and tillering in rice. Plant Cell Physiol 46:1674-1681. doi: 10.1093/pcp/pci183
47. Zhao Y, Hu Y, Dai M, Huang L, Zhou DX (2009) The WUSCHEL-related homeobox gene *WOX11* is required to activate shoot-borne crown root development in rice. Plant Cell 21:736-748. doi: 10.1105/tpc.108.061655
48. Jiang H, Wang S, Dang L, Wang S, Chen H, Wu Y, Jiang X, Wu P (2005) A novel short-root gene encodes a glucosamine-6-phosphate acetyltransferase required for maintaining normal root cell shape in rice. Plant Physiol 138:232-242. doi: 10.1104/pp.104.058248
49. Jia L, Wu Z, Hao X, Carrie C, Zheng L, Whelan J, Wu Y, Wang S, Wu P, Mao C (2011) Identification of a novel mitochondrial protein, short postembryonic roots 1 (SPR1), involved in root development and iron homeostasis in *Oryza sativa*. New Phytol 189:843-855. doi: 10.1111/j.1469-8137.2010.03513.x
50. Woo YM, Park HJ, Su'udi M, Yang JI, Park JJ, Back K, Park YM, An G (2007) Constitutively wilted 1, a member of the rice YUCCA gene family, is required for maintaining water homeostasis and an appropriate root to shoot ratio. Plant Mol Biol 65:125-136. doi: 10.1007/s11103-007-9203-6

51. Kaur R, Singh K, Singh J (2013) A root-specific wall-associated kinase gene, *HvWAK1*, regulates root growth and is highly divergent in barley and other cereals. Funct Integr Genomics 13:167-177. doi: 10.1007/s10142-013-0310-y
52. Hou J, Zheng X, Ren R, Shi Q, Xiao H, Chen Z, Yue M, Wu Y, Hou H, Li L (2022) The histone deacetylase 1/GSK3/SHAGGY-like kinase 2/BRASSINAZOLE-RESISTANT 1 module controls lateral root formation in rice. Plant Physiol 189:858-873. doi: 10.1093/plphys/kiac015
53. Zhu J, Li Y, Lin J, Wu Y, Guo H, Shao Y, Wang F, Wang X, Mo X, Zheng S, Yu H, Mao C (2019) CRD1, an Xpo1 domain protein, regulates miRNA accumulation and crown root development in rice. Plant J 100:328-342. doi: 10.1111/tpj.14445
54. Fusi R, Rosignoli S, Lou H, Sangiorgi G, Bovina R, Pattem JK, Borkar AN, Lombardi M, Forestan C, Milner SG, Davis JL, Lale A, Kirschner GK, Swarup R, Tassinari A, Pandey BK, York LM, Atkinson BS, Sturrock CJ, Mooney SJ, Hochholdinger F, Tucker MR, Himmelbach A, Stein N, Mascher M, Nagel KA, De Gara L, Simmonds J, Uauy C, Tuberosa R, Lynch JP, Yakubov GE, Bennett MJ, Bhosale R, Salvi S (2022) Root angle is controlled by *EGT1* in cereal crops employing an antigravitropic mechanism. Proc Natl Acad Sci USA 119:e2201350119. doi: 10.1073/pnas.2201350119
55. Zhu ZX, Liu Y, Liu SJ, Mao CZ, Wu YR, Wu P (2012) A gain-of-function mutation in *OsIAA11* affects lateral root development in rice. Mol Plant 5:154-161. doi: 10.1093/mp/ssr074
56. Kitomi Y, Inahashi H, Takehisa H, Sato Y, Inukai Y (2012) *OsIAA13*-mediated auxin signaling is involved in lateral root initiation in rice. Plant Sci 190:116-122. doi: 10.1016/j.plantsci.2012.04.005
57. Luo S, Li Q, Liu S, Pinas NM, Tian H, Wang S (2015) Constitutive Expression of *OsIAA9* affects starch granules accumulation and root gravitropic response in *Arabidopsis*. Frontiers of Plant Sciences 6:1156. doi: 10.3389/fpls.2015.01156
58. Nakamura A, Umemura I, Gomi K, Hasegawa Y, Kitano H, Sazuka T, Matsuoka M (2006) Production and characterization of auxin-insensitive rice by overexpression of a mutagenized rice IAA protein. Plant J 46:297-306. doi: 10.1111/j.1365-313X.2006.02693.x
59. Wang L, Guo M, Li Y, Ruan W, Mo X, Wu Z, Sturrock CJ, Yu H, Lu C, Peng J, Mao C (2018) *LARGE ROOT ANGLE1*, encoding *OsPIN2*, is involved in root system architecture in rice. J Exp Bot 69:385-397. doi: 10.1093/jxb/erx427
60. Kang B, Zhang Z, Wang L, Zheng L, Mao W, Li M, Wu Y, Wu P, Mo X (2013) *OsCYP2*, a chaperone involved in degradation of auxin-responsive proteins, plays crucial roles in rice lateral root initiation. Plant J 74:86-97. doi: 10.1111/tpj.12106
61. Ren W, Zhao L, Liang J, Wang L, Chen L, Li P, Liu Z, Li X, Zhang Z, Li J, He K, Zhao Z, Ali F, Mi G, Yan J, Zhang F, Chen F, Yuan L, Pan Q (2022) Genome-wide dissection of changes in maize root system architecture during modern breeding. Nat Plants 8:1408-1422. doi: 10.1038/s41477-022-01274-z
62. Schneider HM, Lor VSN, Hanlon MT, Perkins A, Kaeppler SM, Borkar AN, Bhosale R, Zhang X, Rodriguez J, Bucksch A, Bennett MJ, Brown KM, Lynch JP (2022) Root angle in maize influences nitrogen capture and is regulated by calcineurin B-like protein (CBL)-interacting serine/threonine-protein kinase 15 (*ZmCIPK15*). Plant Cell Environ 45:837-853. doi: 10.1111/pce.14135
63. Suzuki M, Sato Y, Wu S, Kang BH, McCarty DR (2015) Conserved functions of the MATE transporter BIG EMBRYO1 in regulation of lateral organ size and initiation rate. Plant Cell 27:2288-2300. doi: 10.1105/tpc.15.00290

64. Hochholdinger F, Yu P, Marcon C (2018) Genetic control of root system development in maize. Trends Plant Sci 23:79-88. doi: 10.1016/j.tplants.2017.10.004
65. Li F, Yan D, Gao L, Liu P, Zhao G, Jia J, Ren Z (2022b) *TaIAA15* genes regulate plant architecture in wheat. J Integ Agric 21:1243-1252. doi: 10.1016/S2095-3119(20)63480-3
